# Network Pharmacology-Based Investigation into the Mechanisms of Quyushengxin Formula for the Treatment of Ulcerative Colitis

**DOI:** 10.1155/2019/7870424

**Published:** 2019-12-20

**Authors:** Haojie Yang, Ying Li, Sichen Shen, Dan Gan, Changpeng Han, Jiong Wu, Zhenyi Wang

**Affiliations:** Department of Colo-Proctology, Yueyang Hospital of Integrated Traditional Chinese and Western Medicine, Shanghai University of Traditional Chinese Medicine, Shanghai 200437, China

## Abstract

**Objective:**

Ulcerative colitis (UC) is a chronic idiopathic inflammatory bowel disease whose treatment strategies remain unsatisfactory. This study aims to investigate the mechanisms of Quyushengxin formula acting on UC based on network pharmacology.

**Methods:**

Ingredients of the main herbs in Quyushengxin formula were retrieved from the Traditional Chinese Medicine Systems Pharmacology (TCMSP) database. Absorption, distribution, metabolism, and excretion properties of all ingredients were evaluated for screening out candidate bioactive compounds in Quyushengxin formula. Weighted ensemble similarity algorithm was applied for predicting direct targets of bioactive ingredients. Functional enrichment analyses were performed for the targets. In addition, compound-target network, target-disease network, and target-pathway network were established via Cytoscape 3.6.0 software.

**Results:**

A total of 41 bioactive compounds in Quyushengxin formula were selected out from the TCMSP database. These bioactive compounds were predicted to target 94 potential proteins by weighted ensemble similarity algorithm. Functional analysis suggested these targets were closely related with inflammatory- and immune-related biological progresses. Furthermore, the results of compound-target network, target-disease network, and target-pathway network indicated that the therapeutic effects of Quyushengxin on UC may be achieved through the synergistic and additive effects.

**Conclusion:**

Quyushengxin may act on immune and inflammation-related targets to suppress UC progression in a synergistic and additive manner.

## 1. Introduction

Ulcerative colitis (UC) is a chronic and progressive immunologically mediated disease causing consecutive mucosal inflammation of the colon [1, 2]. The onset of UC is most often during young adulthood, which is well characterized by homogeneous and continuous lesions [3]. Although the incidence of UC is increasing in Asia, it is highly diagnosed in the developed countries, especially in Western Europe and North America. Previous reports showed that the overall incidence and prevalence of UC are nearly 1.2/20.3 cases and 7.6/245 per 100,000 persons per year, respectively [4, 5].

UC therapy is aimed to reduce the recurrent rate, as well as improve the life quality and minimize drug-related adverse events. Basic therapies for UC are determined based on the severity of symptoms, which are often thought as step-up approaches. To date, 5-aminosalycilates (5-ASAs) have been the mainstay for treatment of mild-to-moderate UC [6]. Though 5-ASAs are safe and have no dose-related toxicity in short-term use with a dose-response efficacy, long-term use of them might induces adverse events, such as headache, diarrhea, nausea, interstitial nephritis, and hepatitis. In addition, patients with more moderate-to-severe UC after 5-ASAs therapy are typically treated with corticosteroids, and these patients are often followed by transition to a steroid-sparing agent with a thiopurine, adhesion molecule inhibitor, or anti-tumor necrosis factor (TNF) agent [6]. However, these corticosteroid-based therapies also accompany with side effects, such as cataracts, osteopenia, avascular necrosis, insomnia, mood changes, delirium, glaucoma, and adrenal insufficiency [7, 8]. Besides, despite improved medical therapies, it is estimated that about 15% of UC patients still require proctocolectomy [9]. Therefore, it is of great significance to develop more optimized and integrated therapies for UC patients.

To date, an increasing number of traditional Chinese herbal compounds are successfully used for treating UC with less side effects, such as Gegen Qinlian decoction [10], Jianpi Qingchang decoction [11, 12], Zhikang capsule [13], Huangkui Lianchang decoction [14], and Qingchang Wenzhong decoction [15, 16]. Quyushengxin formula is mainly composed of four herbs, *Panax ginseng* C.A. Mey. (Araliaceae), *Astragalus membranaceus* (Fisch) Bunge, *Pulsatilla chinensis* (Bge.) Regel, and *Coptis chinensis* Franch. Our clinical practice demonstrated Quyushengxin formula could relieve the clinical symptoms in active stage and suppress the inflammatory reaction of UC patients and could be used for treating mild-to-moderate UC [17]. Although the therapeutic effects of Quyushengxin on UC are attractive, molecular mechanisms of its action remain to be further elucidated.

Traditional Chinese medicine- (TCM-) oriented network pharmacology provides us a novel way to unveil the molecular mechanisms of TCM through pharmacokinetic evaluation, network/pathway analysis, and target prediction [18, 19]. In this study, we tried to unveil the molecular mechanisms of Quyushengxin formula acting on UC based on network pharmacology.

## 2. Materials and Methods

### 2.1. Screening of Potential Bioactive Compounds in Quyushengxin Formula

Traditional Chinese Medicine Systems Pharmacology Database (TCMSP, http://lsp.nwu.edu.cn) is a systems pharmacology platform of Chinese herbal medicines that captures the relationships between drugs, targets, and diseases [20]. Ingredients along with their molecular weight (MW), water partition coefficient (AlogP), number of hydrogen bond donors (Hodn), number of hydrogen acceptors (Hacc), oral bioavailability (OB), Caco-2 permeability (Caco-2), blood-brain barrier (BBB), drug-likeness (DL), fractional negative accessible surface area (FASA) ,and half-life (HL), of all four herbs in Quyushengxin formula were retrieved from TCMSP. Then, absorption, distribution, metabolism, and excretion (ADME) properties, including OB, DL, and HL, were evaluated for screening out bioactive compounds. The potential bioactive compounds in Quyushengxin were predicted and sifted out via an integrated model including PreOB (for prediction of OL), PreDL (for prediction of DL), and PreHL (for prediction of HL) [21, 22]. In detail, OB value was obtained by OBioavail 1.1, and the compounds with OB ≥ 30% were selected out for further analysis [20, 23]. PreDL was utilized to calculate the DL index of compounds, and compounds with DL ≥ 0.18 were included for further research. The DL evaluation approach was constructed via both Tanimoto coefficient and molecular descriptors, and the formula is listed as follows:

(1) $$\begin{array}{c} T\left(X,Y\right)=\frac{X\cdot Y}{{\left|X\right|}^{2}+{\left|Y\right|}^{2}-X\cdot Y}, \end{array}$$

where *X* was the molecular descriptors of herbal ingredients and *Y* showed the average molecular properties of all molecules in the DrugBank database (http://www.drugbank.ca/).

Besides, PreHL was estimated by combining multivariable linear regression model and MLR (mixed logistic regression) algorithm [22], as follows:

(2) $$\begin{array}{c} Y\left(t_{1/2}\right)=13.310\left(\pm 13.31\right)+13.376\left(\pm 13.37\right)\times \text{nArCO}\\ +7.092\left(\pm \text{nA}7\right)\times \text{H}7\text{m}+0.053\left(\pm 0.007\right)\times \left(\text{D}/\text{Dr}09\right)+19.377\left(\pm 4.052\right)\\ \times N-070-7.598\left(\pm 70-7.\right)\times C-033-347.423\left(\pm 33-347.\right)\\ \times \text{JGI}6+32.752\left(\pm \text{JG}2\right)\times \text{nRC}=N-0.100\left(\pm \text{nR0}\right)\times \text{Mor}02\text{e},\\ R^{2}=0.65,\\ Q^{2}=0.62,\\ F=27.272,\\ \text{SEE}=8.127,\\ N_{\text{training}}=126,\\ N_{\text{test}}=43, \end{array}$$

where *R*^2^ was the correlation coefficient of training set and *Q*^2^ was the correlation coefficient of external test sets of the model. SEE was the estimated standard deviation of training set. *F* was the mean square ratio. Besides, *N*_training_ indicated the number of chemical compounds in the training set, and *N*_test_ indicated the number of chemical compounds in the test set. It was evidenced that there were eight descriptors satisfying the linear regression as follows: nArCO, H7m, D/Dr09, N-070, C-032, JGI6, nRC=N, and Mor02e. Finally, 4 ≤ HL ≤ 8 was defined as appropriate selection criteria for drug HL evaluation.

### 2.2. Prediction of the Candidate Targets of Bioactive Compounds

Weighted ensemble similarity (WES) algorithm was applied for predicting direct targets of the bioactive compounds via a large scale of drug target relationships [24]. Those targets with likelihood score ≥7 were deemed as direct targets in this study. Thereafter, candidate targets were mapped to Uniprot (http://www.uniprot.org/) for annotation and normalization.

### 2.3. Functional Enrichment Analyses

Gene Ontology- (GO-) biological processes (BPs) and Kyoto Encyclopedia of Genes and Genomes (KEGG, http://www.genome.jp/kegg/) pathways of the candidate targets of bioactive compounds were predicted via the Database for Annotation, Visualization, and Integrated Discovery (DAVID) database [25] with *P* < 0.05 as the criterion for significance.

### 2.4. Prediction of Target-Related Disease

Target-related diseases were predicted by integrating multisource databases, including Comparative Toxicogenomics Database (CTD, http://ctdbase.org/) [26], Therapeutic Target Database (TTD, http://bidd.nus.edu.sg/group/cjttd/) [27], and PharmGKB database (https://www.pharmgkb.org/) [28].

### 2.5. Network Construction

Three kinds of networks in this study were established using Cytoscape 3.6.0 software [29]: compound-target network (C-T network), target-disease network (T-D network), and target-pathway network (T-P network). C-T network was composed of bioactive compounds and their potential targets, which was built to reveal the drug-target interactions. T-D network was built based on the potential targets and their related diseases. The pathway information of targets was selected from the results for KEGG pathway enrichment analysis by excluding those pathways with no relevance to UC based the latest pathological information of UC. T-P network was generated based on potential targets and UC-related pathways. In the networks, the nodes represented compounds, targets, diseases, and pathways, and the edges displayed the interactions between two nodes. Furthermore, the significance of each node in the networks was assessed via one crucial topological parameter, namely, “degree,” which was defined as the total of edges related with a node [30, 31]. Degree of all nodes was analyzed using plugin NetworkAnalyzer of Cytoscape 3.6.0.

## 3. Results

### 3.1. Screening of Potential Bioactive Compounds from Four Herbs in Quyushengxin Formula

Quyushengxin formula consists of 4 main herbs: *Panax ginseng* C.A. Mey. (Araliaceae), *Astragalus membranaceus* (Fisch) Bunge, *Pulsatilla chinensis* (Bge.) Regel, and *Coptis chinensis* Franch. After retrieving from TCMSP, 190, 87, 57, and 48 ingredients were obtained for these four herbs, respectively. Based on the criteria of OB ≥ 30%, DL ≥ 0.18, and 4 ≤ HL ≤ 8, 41 potential bioactive compounds, including quercetin, ursolic acid, kaempferol, *β*-sitosterol, and rutin, were sifted out (Table 1), which accounted for 10.73% of all 382 ingredients in Quyushengxin.

### 3.2. Establishment of C-T Network

**Figure 1:**
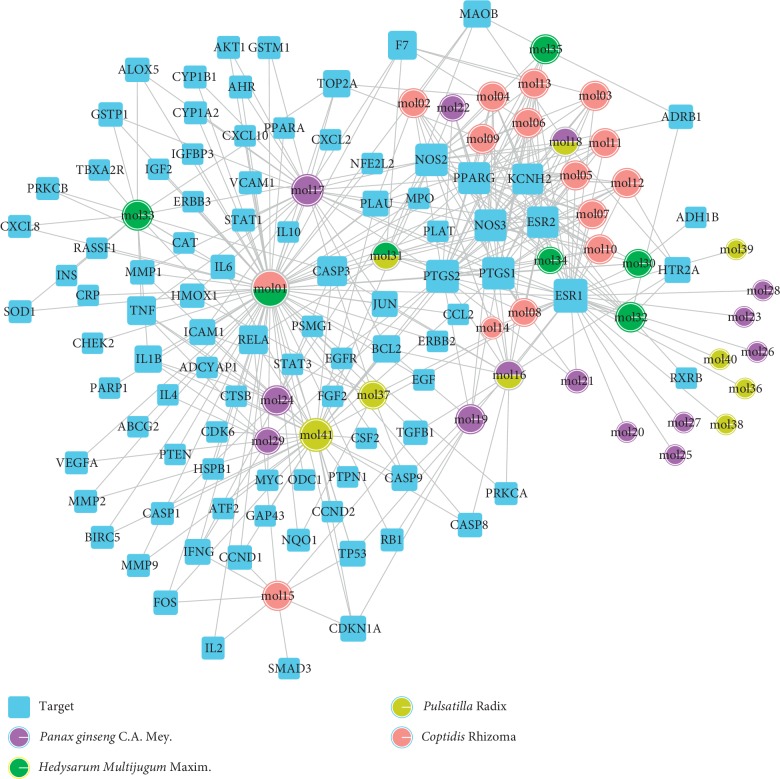
Compound-target network. A compound node and a target node are connected.

Candidate targets of the 41 bioactive compounds were predicted via WES algorithm. A total of 367 potential targets for these 41 bioactive compounds were obtained. After removing the overlapping targets, 94 candidate proteins were reserved. Then, C-T network was built by Cytoscape 3.6.0 which contains 367 connections between 41 compounds and corresponding 94 candidate targets (Figure 1). The degrees of the 41 bioactive compounds in the C-T network were calculated and are displayed in Table 1. The average degree of targets per compound was 4.7, indicating multitarget functions of Quyushengxin formula. Among the 41 bioactive compounds, 8 of them showed a high degree (degree > 10). Quercetin possessed the highest degree of targets (degree = 73), followed by ursolic acid (degree = 35), kaempferol (degree = 26), *β*-sitosterol (degree = 15), rutin (degree = 15), 7-O-methylisomucronulatol (degree = 11), stigmasterol (degree = 10), and isorhamnetin (degree = 10).

The degree of the candidate targets was also calculated and displayed in Table 2. Eight out of the 94 compounds possessed a degree larger than 10, including ESR1 (estrogen receptor 1, degree = 34), PTGS2 (prostaglandin-endoperoxide synthase 2, degree = 27), NOS2 (nitric oxide synthase 2, degree = 25), PTGS1 (degree = 23), PPARG (peroxisome proliferator-activated receptor gamma, degree = 21), NOS3 (degree = 21), ESR2 (degree = 17), and KCNH2 (Potassium Voltage-Gated Channel Subfamily H Member 2, degree = 13).

### 3.3. GO-BP Analysis

**Figure 2:**
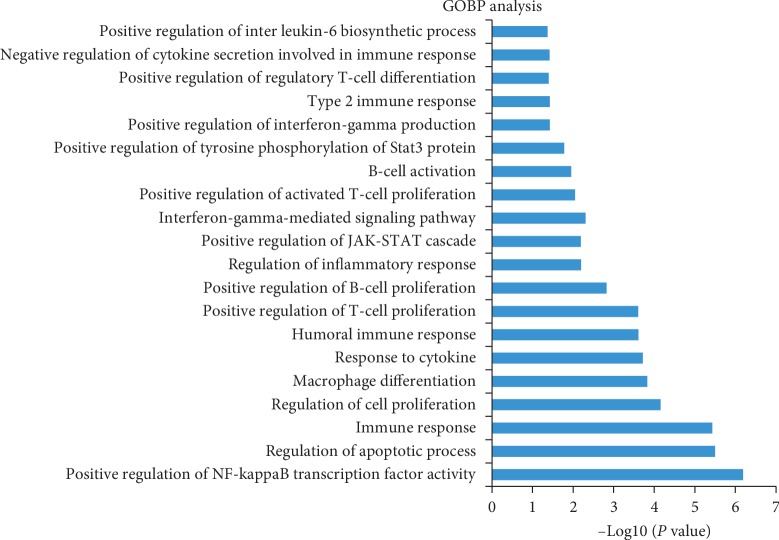
Gene Ontology biological process analysis. The *y*-axis shows significantly enriched “Biological Processes” categories, and the *x*-axis shows the enrichment scores of these terms.

To further validate whether biological processes enriched by candidate targets as mentioned above were correlated with UC, GO-BP enrichment analysis was performed via DAVID. The top 20 significant GO-BP terms are shown in Figure 2. Most of them were strongly associated with inflammatory- and immune-related BPs such as “positive regulation of interleukin-6 biosynthetic process,” “regulation of inflammatory response,” “immune response,” and “positive regulation of T-cell proliferation.” In short, the 41 bioactive compounds in Quyushengxin formula may act on 94 candidate targets with inflammatory- and immune-related effects to affect UC pathogenesis.

### 3.4. Establishment of T-D Network

**Figure 3:**
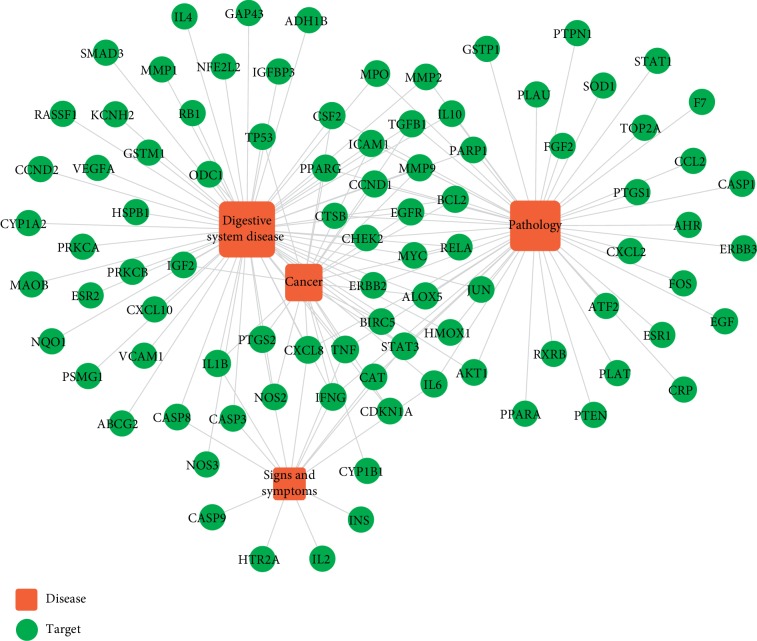
Target-disease network. Red square represents disease and green circle represents target.

Target-related diseases were predicted by mapping them to integrating multisource databases, including CTD, TTD, and PharmGKB. A T-D network consisting of 90 targets and 4 kinds of diseases was built (Figure 3). The four diseases were digestive system disease (degree = 60), pathology (degree = 49), cancer (degree = 23), and signs and symptoms (degree = 14).

### 3.5. T-P Network Evaluation

**Figure 4:**
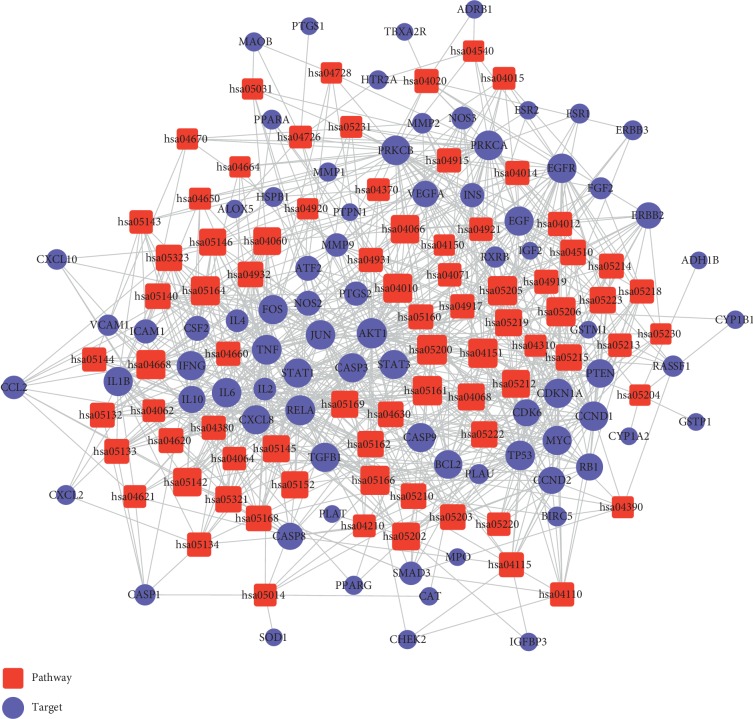
Target-pathway network. Red square represents pathway and purple circle represents targets.

KEGG pathway enrichment analysis was performed for the 94 targets, and T-P network was built. Results displayed that 79 targets could be further mapped to 78 pathways, including “mTOR signaling pathway,” “T-cell receptor signaling pathway,” “JAK-STAT signaling pathway,” and “FOXO signaling pathway” (Figure 4). The average degree of targets was 6.85, and the average degree of pathway was 2.8. In addition, 71 candidate targets could be mapped to several pathways (≥5), suggesting that these targets might mediate the cross-talk and interactions between different pathways. Besides, those pathways (70/78) mapped by multiple targets (≥8) might be the main factors for UC development and progression. These pathways were further divided into five function modules, including inflammatory regulation, immune regulation, metabolic regulation, bacterial infection or mycosis and other function.

### 3.6. Establishment of Compound-Target-Function Module Network

**Figure 5:**
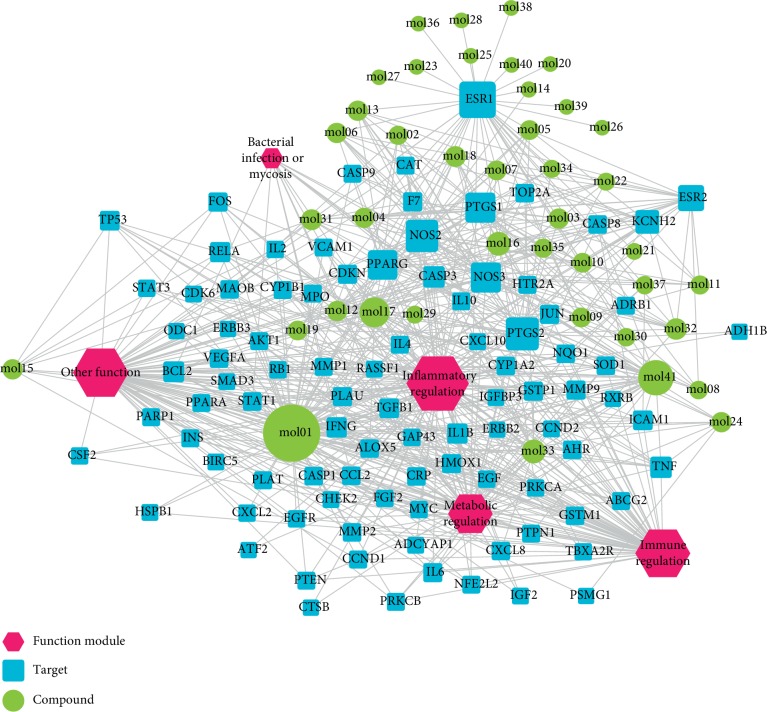
Compound-target-function module network. Green circle represents compound, blue square represents target, and red hexagon represents function module.

By combing the networks above, a compound-target-function module network was built, which included 140 nodes (5 function modules, 41 compounds and 95 targets) and 653 edges (Figure 5).

### 3.7. Details of 4 UC-Related Pathways from T-P Network Analysis

**Figure 6:**
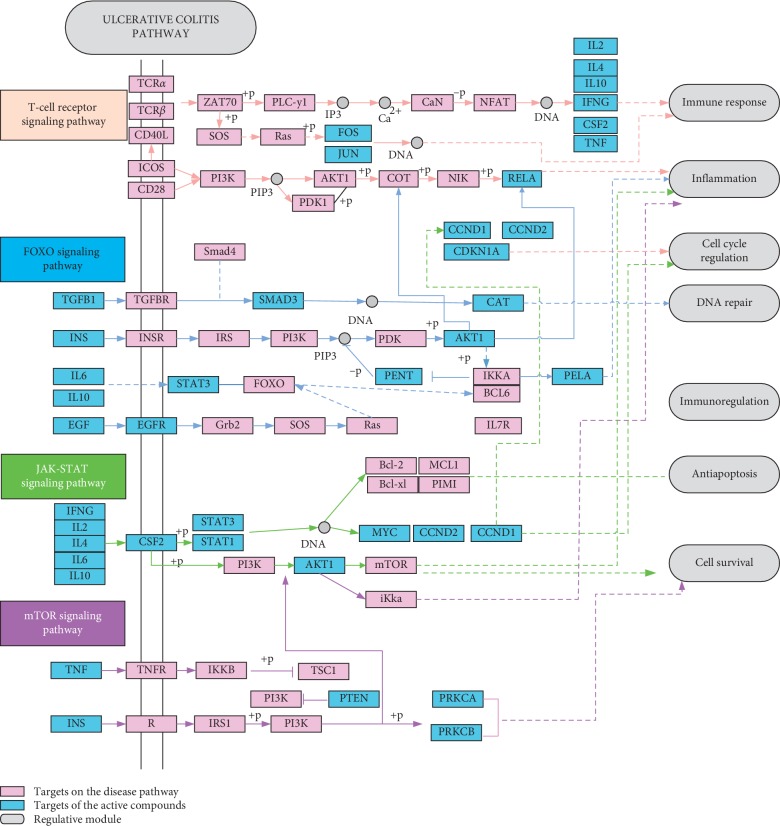
Distribution of targets of Quyushengxin formula in the “UC-related pathway.” Arrow shows activation effect; T-shaped arrow shows inhibition effect, and dotted arrow represents indirect activation effect or inhibition effect.

To further unveil the multi-targets mechanisms of Quyushengxin formula in the treatment of UC, an integrated “UC-related pathway” was established according to the key pathways from the T-P network analysis. UC-related pathways as shown in Figure 6 were composed of four pathways, including “T cell receptor signaling pathway” (hsa04660), “FOXO signaling pathway” (hsa04068), “JAK-STAT signaling pathway” (hsa04630) and “mTOR signaling pathway” (hsa04150). Those targets of the integrated “UC-related pathways” displayed the functional relationship with the UC-related proteins. UC-related pathways can be divided into three modules: immunology module, metabolism module and cell apoptosis-related module. Immunology module consisted of “T cell receptor signaling pathway” (hsa04660), and metabolism module consisted of “FOXO signaling pathway” (hsa04068). Cell apoptosis-related module was comprised of “JAK-STAT signaling pathway” (hsa04630) and “mTOR signaling pathway” (hsa04150). Taken together, Quyushengxin formula may well regulate immunology progress, metabolism progress and cell apoptosis progress to suppress UC progression.

## 4. Discussion

TCM has the advantages of high treatment efficacy and low treatment cost and side effect in the treatment of several diseases, including UC in China for several thousands of years [32–34]. After preliminary screening based on ADME properties, 41 potential bioactive compounds of Quyushengxin were screened out. Thereafter, 94 candidate targets of these 41 bioactive compounds were predicted for further analysis. Functional enrichment analyses suggested that these targets were closely related with inflammatory- and immune-related biological processes. Besides, a C-T network, a T-D network, a T-P network, and a compound-target-function module network were built. These networks indicated that the therapeutic effects of Quyushengxin on UC may be achieved through the synergistic and additive effects on multiple molecules and multiple pathways with immune and inflammatory effects to treat UC.

Previous reports showed that the TCMSP-based method was reliable for screening out bioactive compounds of TCM for treatment of thrombosis [35], gastric precancerous lesions [36], cardiocerebrovascular disease [37], and rheumatoid arthritis [38]. In this study, 41 bioactive compounds of Quyushengxin formula were selected out by using TCMSP database in combination with ADME properties. Most of the 41 compounds have been reported to have anti-inflammatory and immune-regulatory effects. For example, quercetin (mol01, OB = 46.43%, DL = 0.28, HL = 14.40) could inhibit lipopolysaccharide- (LPS-) induced interleukin- (IL-) 6 production [39], TNF-*α* production, and IL-8 production [40, 41] to exert anti-inflammatory effect. Besides, ursolic acid (mol17, OB = 16.77%, DL = 0.75, HL = 5.28) was reported to have human neutrophil elastase inhibitory effect both *in vitro* and *in vivo* [42]. Kaempferol (mol17, OB = 41.88%, DL = 0.24, HL = 14.74) was reported to significantly reduce the overproduction of TNF-*α*, IL-1*β*, IL-6, intercellular adhesion molecule- (ICAM-) 1, and vascular cell adhesion molecule- (VCAM-) 1 induced by LPS [43]. In addition, *β*-sitosterol (mol16, OB = 36.91%, DL = 0.75, HL = 5.36) and rutin (mol33, OB = 3.2%, DL = 0.68, HL = 6.22) were shared with significant anti-inflammatory activity [44, 45]. Above all, TCMSP-based systems pharmacology sifted out 41 potential bioactive compounds in Quyushengxin formula for treatment of UC.

Eight of the 94 targets have degree larger than 10 in the C-T network, including ESR1, PTGS2, NOS2, PTGS1, PPARG, NOS3, ESR2, and KCNH2. ESR1 was targeted by 34 compounds, which contributed to T-cell-mediated autoimmune inflammation by promoting T-cell activation and proliferation [46]. Besides, PTGS2 with the second highest degree played a critical role in the pathogenesis of gut inflammation [47, 48]. Moreover, PPARG was demonstrated to be able to downregulate proinflammatory cytokines production, such as IL-4, -5, and -6. In addition, PPARG could also enable to interfere with profibrotic molecules, such as platelet-derived growth factor (PDGF), IL-1, and transforming growth factor beta (TGF-*β*) [49]. These results suggested that Quyushengxin formula could probably treat UC by regulating anti-inflammatory action and the immune system.

In this study, 94 targets were utilized to perform T-P network analysis, and the results showed that 79 targets could be further mapped to 78 pathways. Meanwhile, numerous pathways mapped by multiple targets might be the main factors for UC progression. Four pathways including “T-cell receptor signaling pathway,” “FOXO signaling pathway,” “JAK-STAT signaling pathway,” and “mTOR signaling pathway” were closely associated with immune and inflammatory effects. T-cell receptors play significant role in function of T cells and formation of the immunological synapse, and they connected T cells and the antigen-presenting cells [50]. T-cell receptor pathway was reported to be important in regulation of UC [51, 52]. FOXO pathway plays a key role in regulating the expression of genes related to cell function such as apoptosis, cell cycle, oxidative stress, and differentiation [53–55]. FOXO3a was shown to control the inflammatory response and help maintain the homeostasis of the intestinal mucosa, which may also be a protective factor in the gut, and maintain a balance between the mucosal immune hemostasis against intravascular bacteria and inflammatory cytokines [56]. Besides, JAK-STAT pathway is the fulcrum for many important cellular processes, including cell survival, differentiation, proliferation, and regulation of immune function [57]. The mTOR pathway plays an important role in regulation of cell metabolism, proliferation, and autophagy. It is reported that mTOR signaling pathway was activated in bacteria-induced colitis in mice [58]. Inhibitors of mTOR signaling pathway are effective as anti-inflammatory drugs in treating colitis [59–61]. Therefore, Quyushengxin might suppress UC progression through targeting these anti-inflammation, autophagy, and immunoregulation pathways.

Nevertheless, limitations in this study could never be neglected. First, results in this study were mainly based on known chemical components in Quyushengxin, related targets, and pathways in UC. With the development of science and technology, new components in Quyushengxin, as well as new targets and pathways in UC will be further discovered, which will supply us with more theoretical evidences for further elucidation of underlying mechanisms of UC pathology. Second, the interaction relationships of the nodes in the networks, such as the action type, e.g., activation, inhibition, binding, and catalysis, and the action effect, e.g., positive, negative, and unspecified, are not investigated due to lack of these data. Third, due to the complex interaction between TCM and the human body, many of its acting mechanisms still needed to be further elucidated via pharmacokinetic test and other experiments.

## 5. Conclusion

In short, network pharmacology analysis of Quyushengxin showed that 41 bioactive components of Quyushengxin may act on 94 immune and inflammation-related targets to suppress UC progression in a synergistic and additive manner, which may provide us with a new starting point for a more detailed knowledge of mechanisms of UC pathogenesis.

## Figures and Tables

**Table 1:** Details of 41 bioactive compounds and their biological parameters.

ID	Compounds	Structure	OB (%)	DL	HL	Degree	Herb
mol01	Quercetin	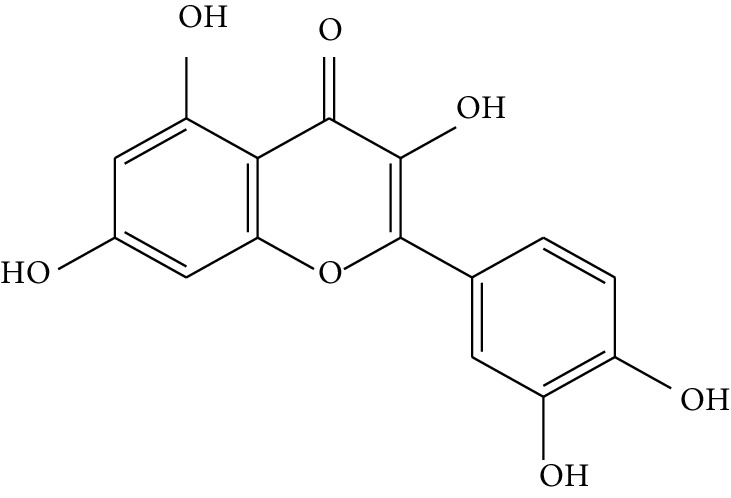	46.43	0.28	14.40	73	*Coptis chinensis* Franch *Astragalus membranaceus* (Fisch) Bunge

mol02	Ferulic acid	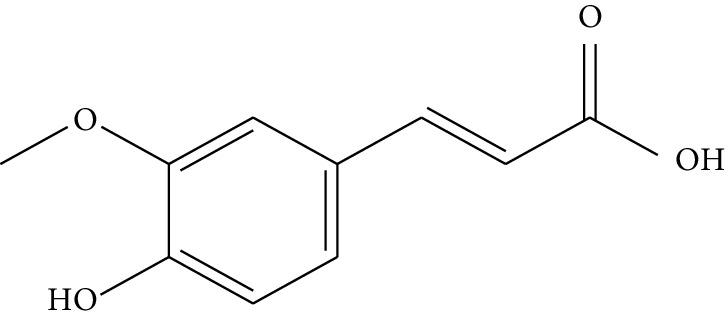	39.56	0.06	2.38	7	*Coptis chinensis* Franch

mol03	Palmatine	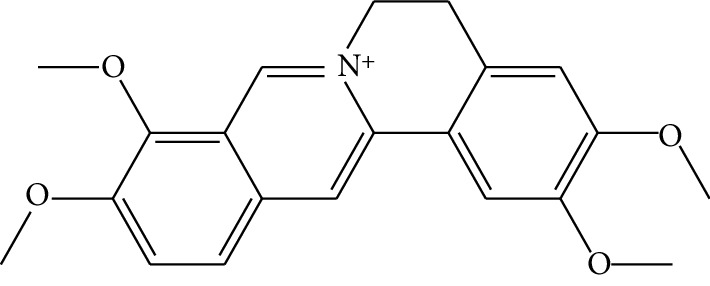	64.60	0.65	2.25	9	*Coptis chinensis* Franch

mol04	Jatrorrizine	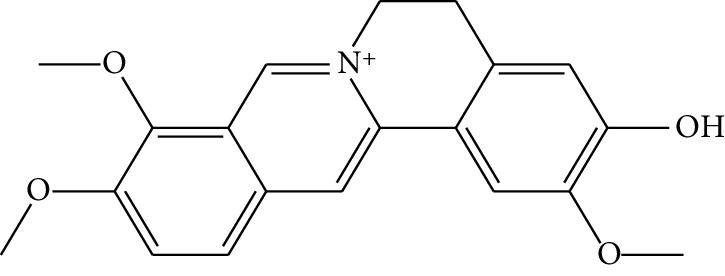	19.65	0.59	4.21	9	*Coptis chinensis* Franch

mol05	Berberine	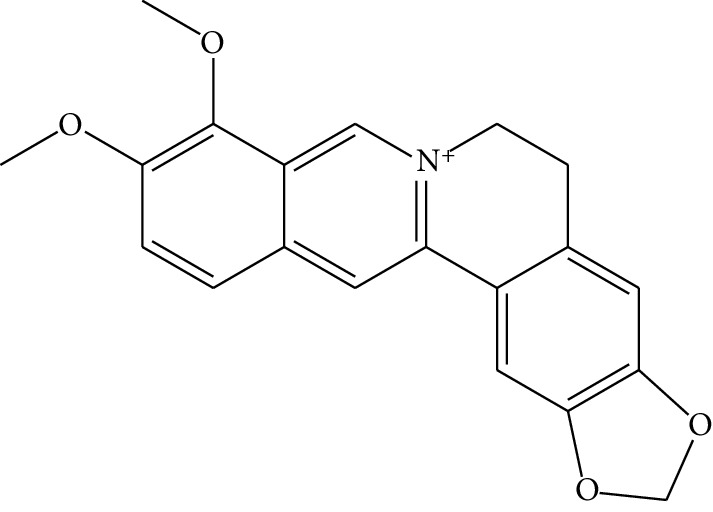	36.86	0.78	6.57	8	*Coptis chinensis* Franch
mol06	Columbamine	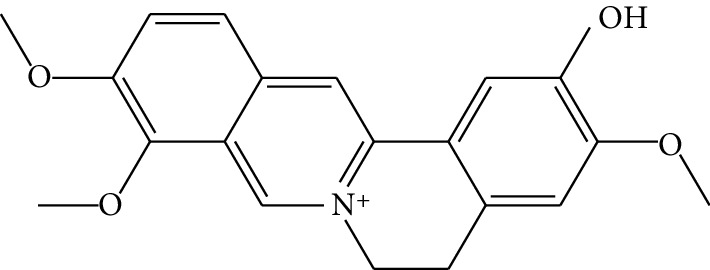	26.94	0.59	5.21	9	*Coptis chinensis* Franch

mol07	Coptisine	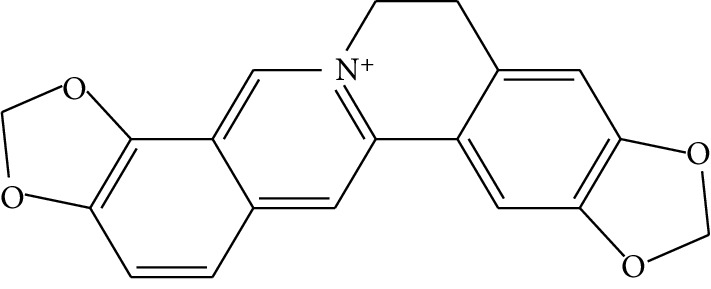	30.67	0.86	9.33	8	*Coptis chinensis* Franch

mol08	Worenine	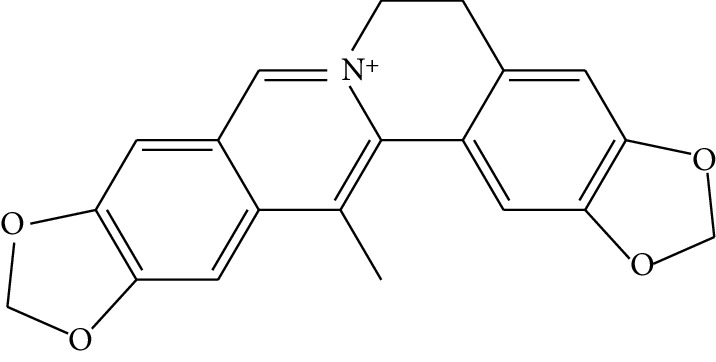	45.83	0.87	8.41	6	*Coptis chinensis* Franch

mol09	Magnoflorine	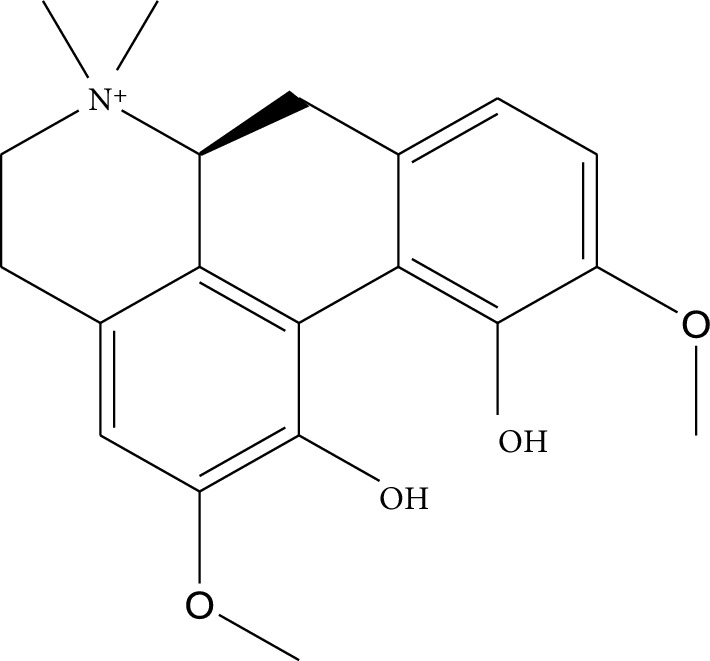	0.48	0.55	6.22	8	*Coptis chinensis* Franch

mol10	Berberrubine	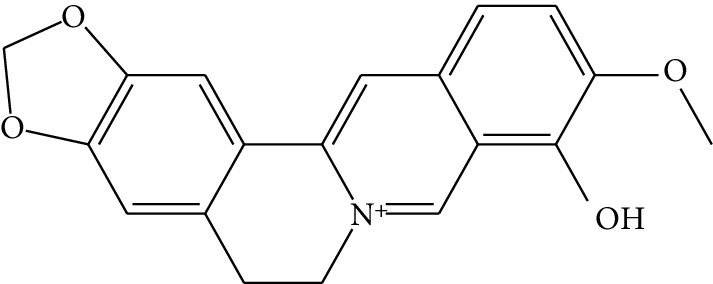	35.74	0.73	6.46	8	*Coptis chinensis* Franch
mol11	Epiberberine	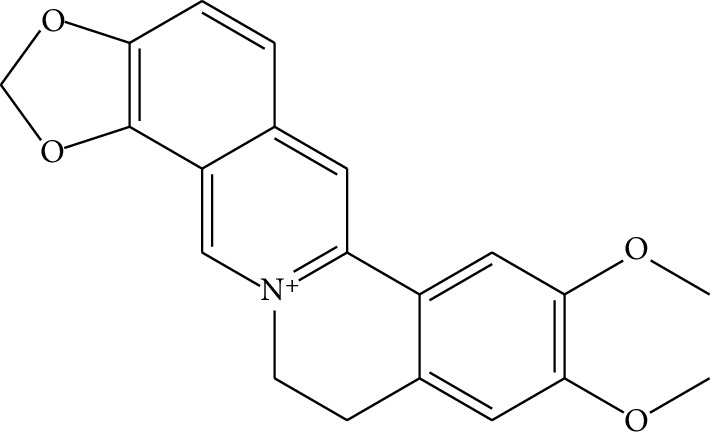	43.09	0.78	6.10	7	*Coptis chinensis* Franch

mol12	(*R*)-Canadine	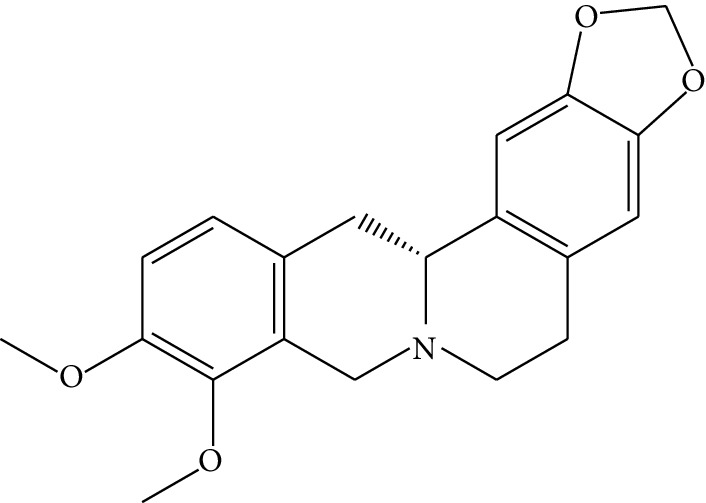	55.37	0.77	6.41	9	*Coptis chinensis* Franch

mol13	Berlambine	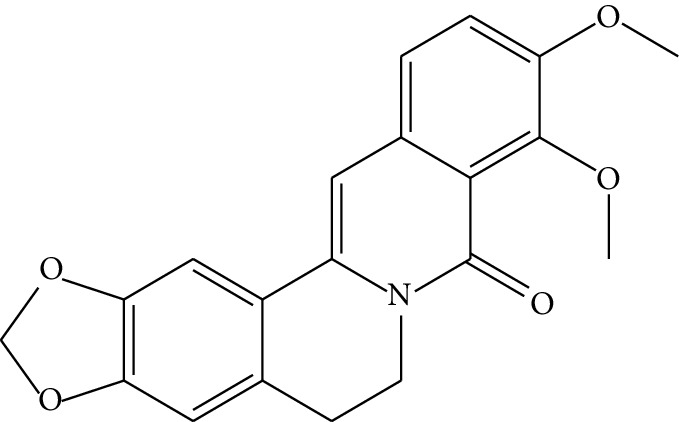	36.68	0.82	7.33	9	*Coptis chinensis* Franch

mol14	Corchoroside A_qt	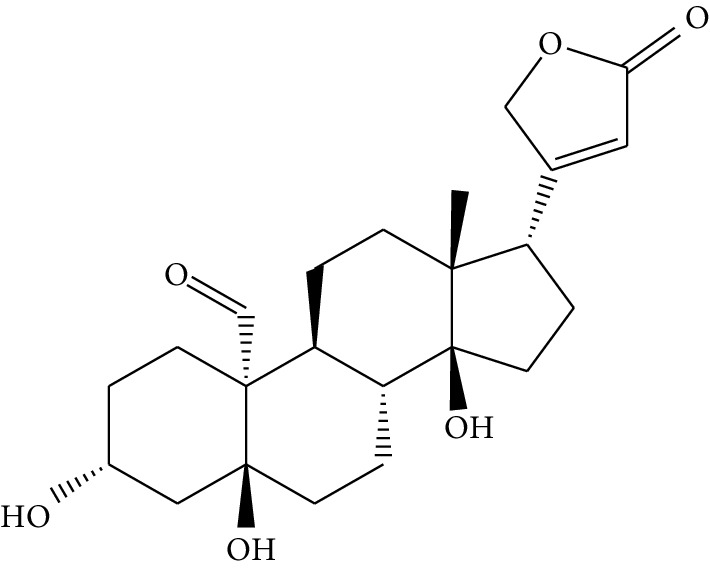	104.95	0.78	6.68	2	*Coptis chinensis* Franch
mol15	Tetrandrine	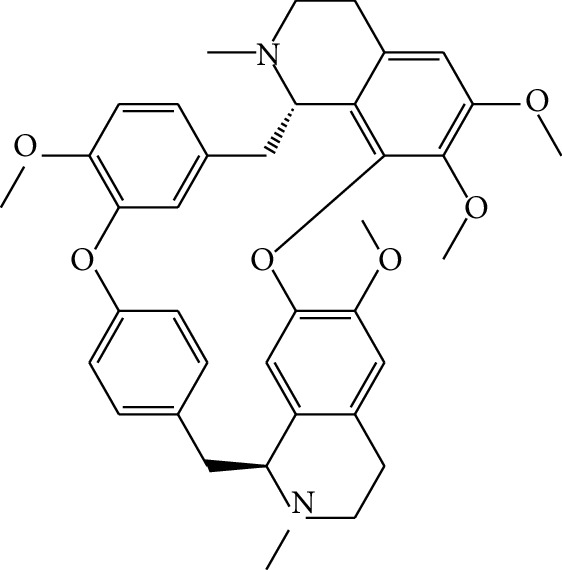	26.64	0.10	4.77	9	*Coptis chinensis* Franch

mol16	*β*-Sitosterol	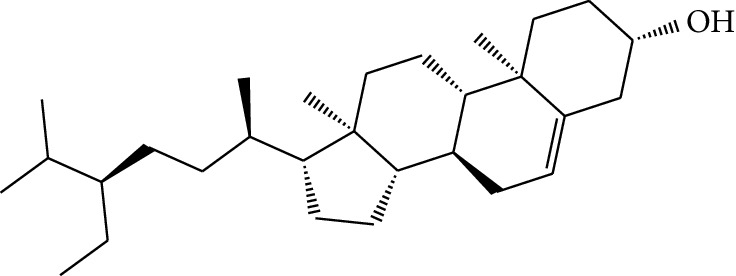	36.91	0.75	5.36	15	*Panax ginseng* C.A. Mey. (Araliaceae) *Pulsatilla chinensis* (Bge.) Regel

mol17	Kaempferol	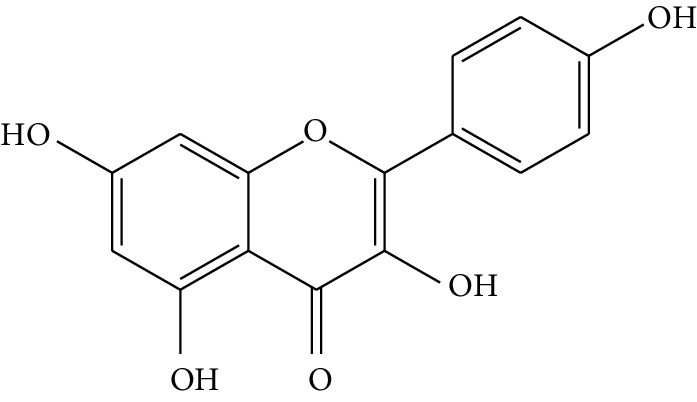	41.88	0.24	14.74	26	*Panax ginseng* C.A. Mey. (Araliaceae)

mol18	Stigmasterol	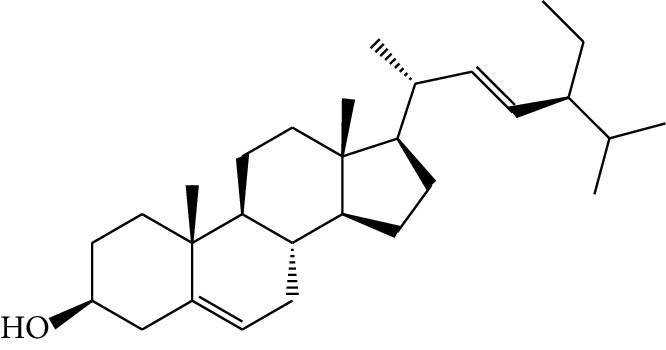	43.83	0.76	5.57	10	*Panax ginseng C.A. Mey*. (*Araliaceae*)G *Pulsatilla chinensis* (Bge.) Regel

mol19	*β*-Elemene	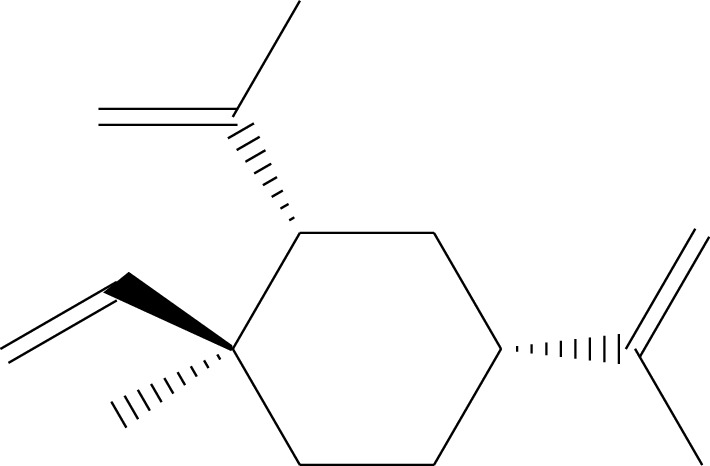	25.63	0.06	6.32	8	*Panax ginseng* C.A. Mey. (Araliaceae)
mol20	Ginsenoside Ro_qt	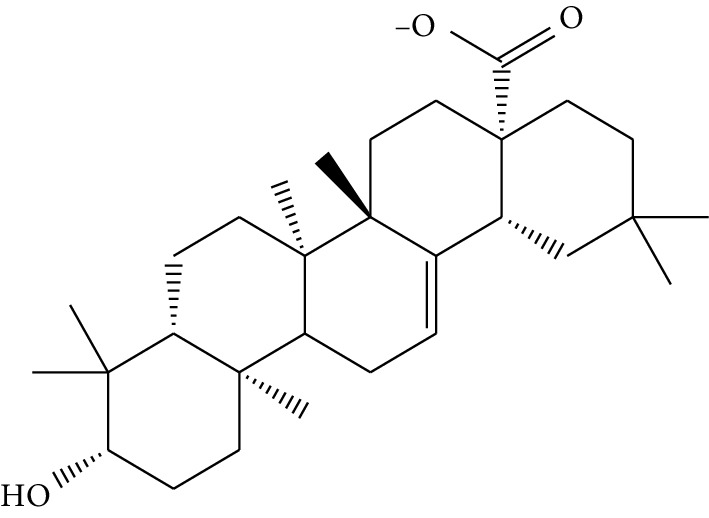	17.62	0.76	7.50	1	*Panax ginseng* C.A. Mey. (Araliaceae)

mol21	Dianthramine	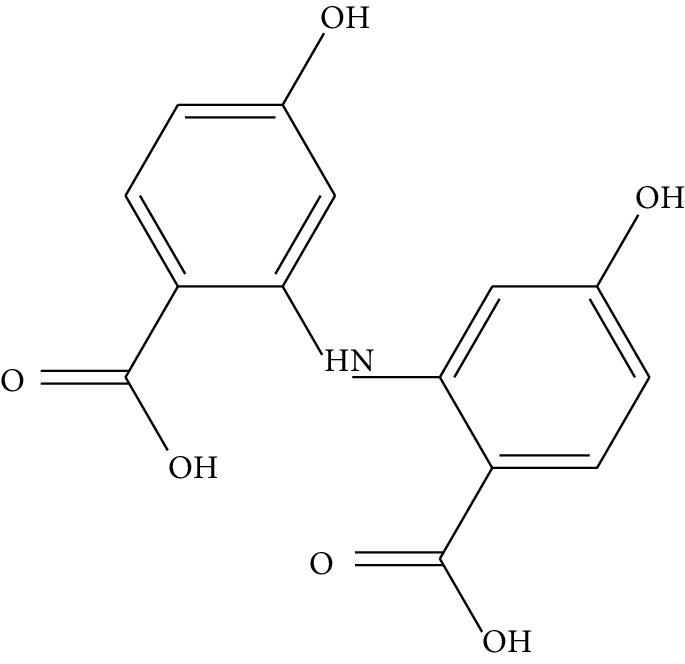	40.45	0.20	5.14	3	*Panax ginseng* C.A. Mey. (Araliaceae)

mol22	Arachidonate	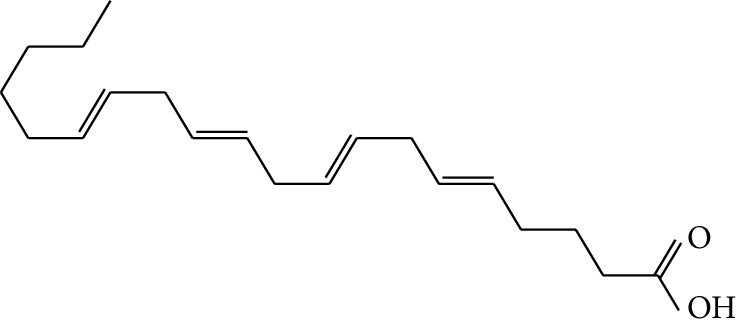	45.57	0.20	7.56	5	*Panax ginseng* C.A. Mey. (Araliaceae)

mol23	Ginsenoside La_qt	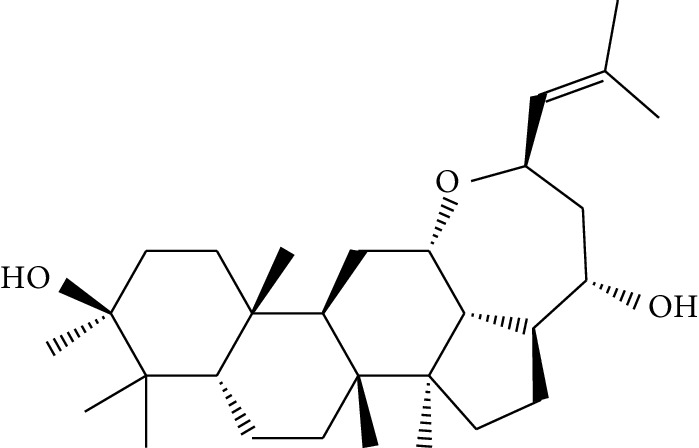	15.70	0.78	5.20	1	*Panax ginseng* C.A. Mey. (Araliaceae)
mol24	Ginsenoside rh2	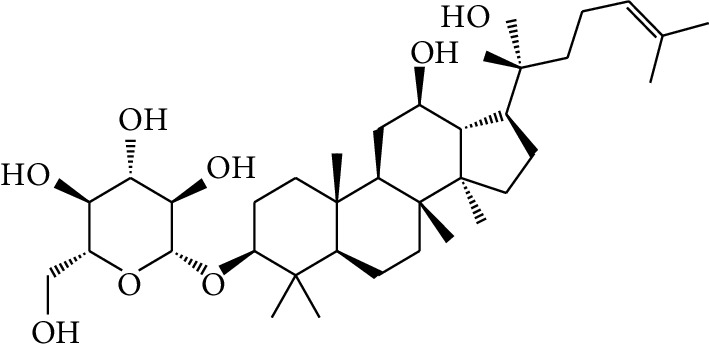	36.32	0.56	11.08	9	*Panax ginseng* C.A. Mey. (Araliaceae)

mol25	Ginsenoside-Rh3_qt	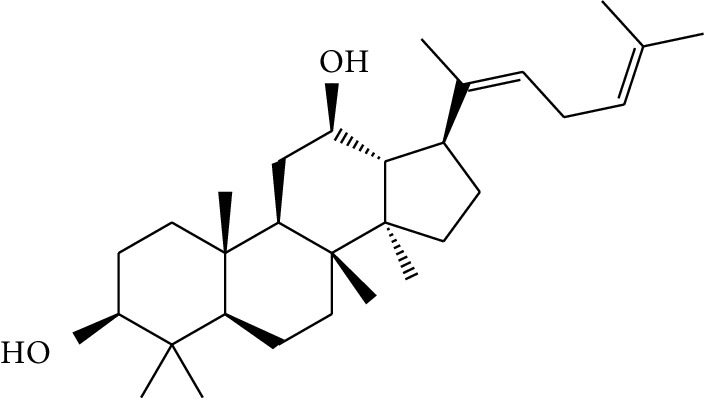	13.09	0.76	6.22	1	*Panax ginseng* C.A. Mey. (Araliaceae)

mol26	Ginsenoside-Rh4_qt	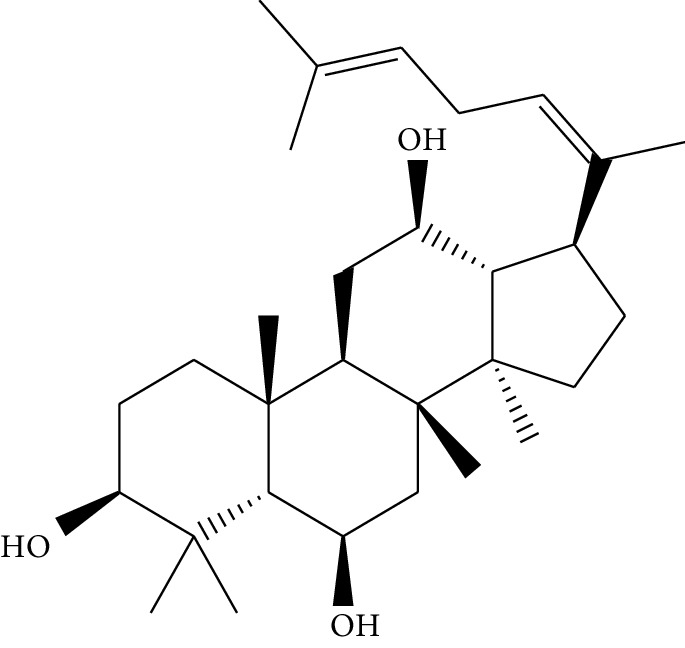	31.11	0.78	6.97	1	*Panax ginseng* C.A. Mey. (Araliaceae)

mol27	Malkangunin	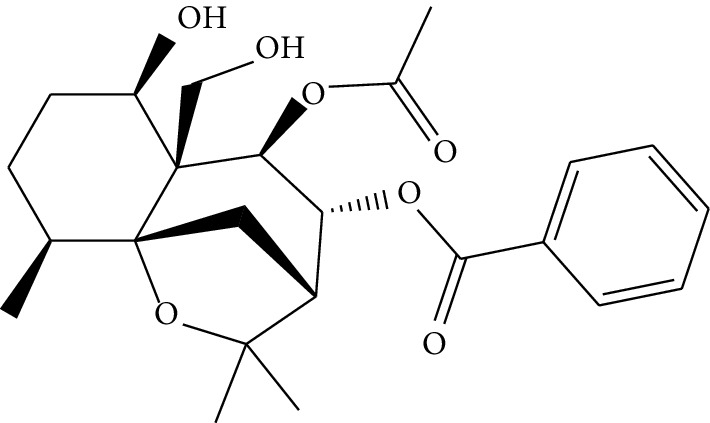	57.71	0.63	4.09	1	*Panax ginseng* C.A. Mey. (Araliaceae)
mol28	Alexandrin_qt	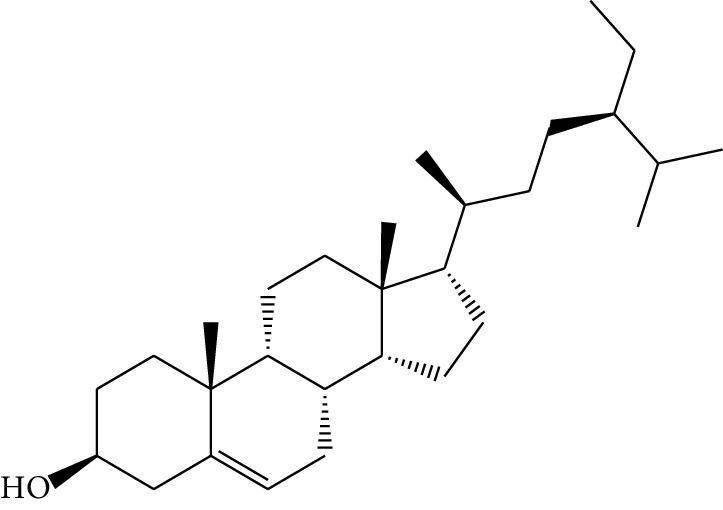	36.91	0.75	5.53	1	*Panax ginseng* C.A. Mey. (Araliaceae)

mol29	Ginsenoside rf	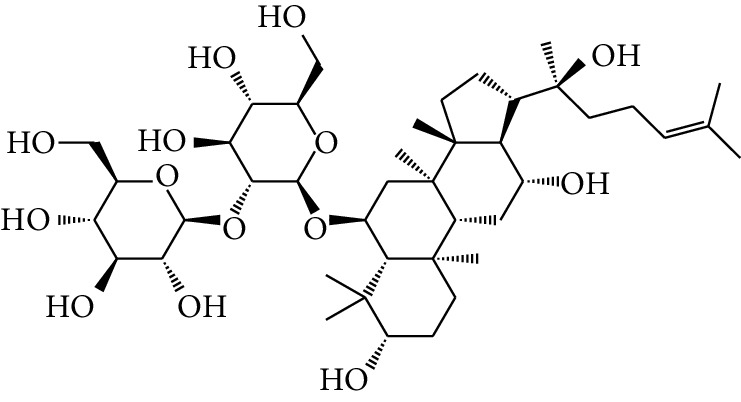	17.74	0.24	4.66	5	*Panax ginseng* C.A. Mey. (Araliaceae)

mol30	Hederagenin	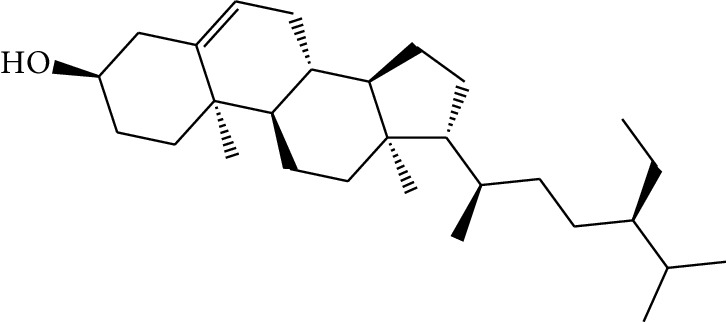	36.91	0.75	5.35	6	*Astragalus membranaceus* (Fisch) Bunge

mol31	Isorhamnetin	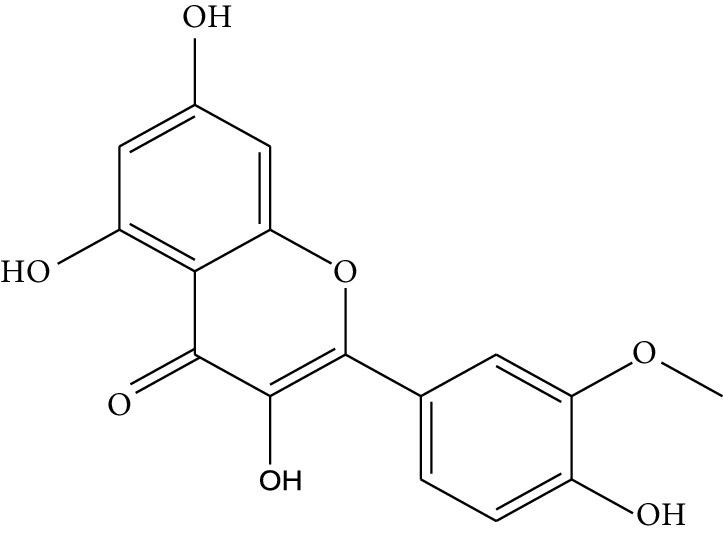	49.60	0.31	14.34	10	*Astragalus membranaceus* (Fisch) Bunge *Pulsatilla chinensis* (Bge.) Regel
mol32	7-O-methylisomucronulatol	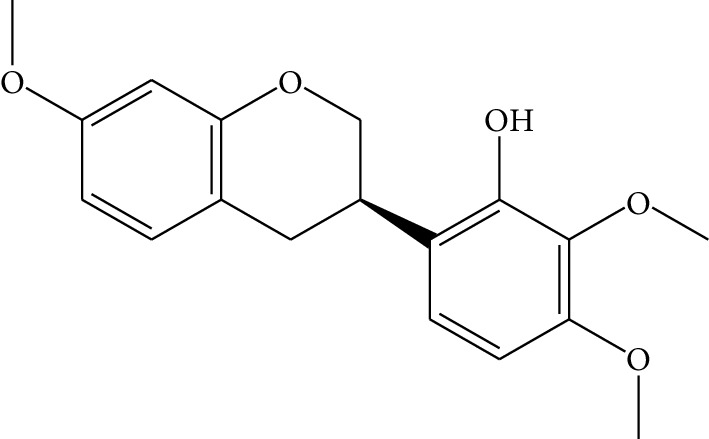	74.69	0.30	2.98	11	*Astragalus membranaceus* (Fisch) Bunge

mol33	Rutin	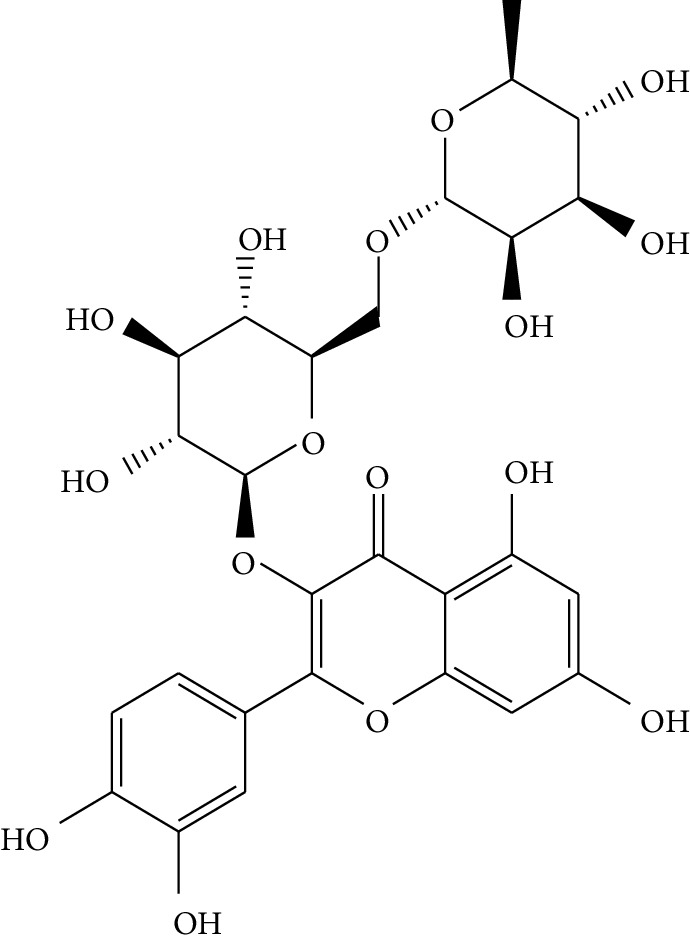	3.20	0.68	6.22	15	*Astragalus membranaceus* (Fisch) Bunge

mol34	1,7-Dihydroxy-3,9-dimethoxy pterocarpene	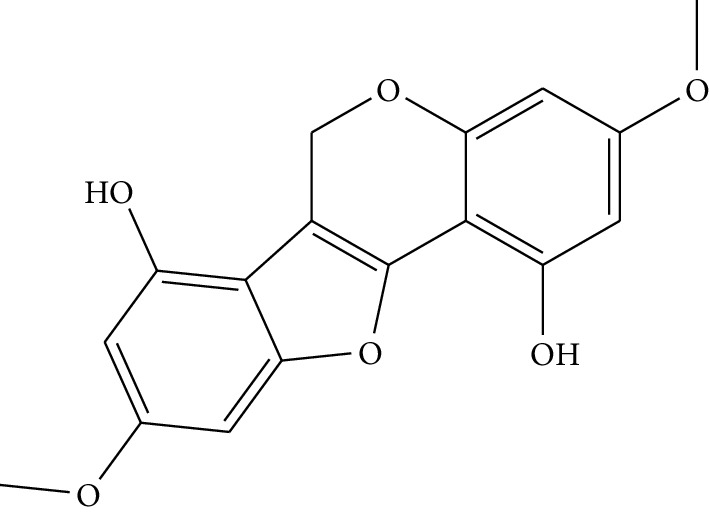	39.05	0.48	7.95	5	*Astragalus membranaceus* (Fisch) Bunge

mol35	Isoferulic acid	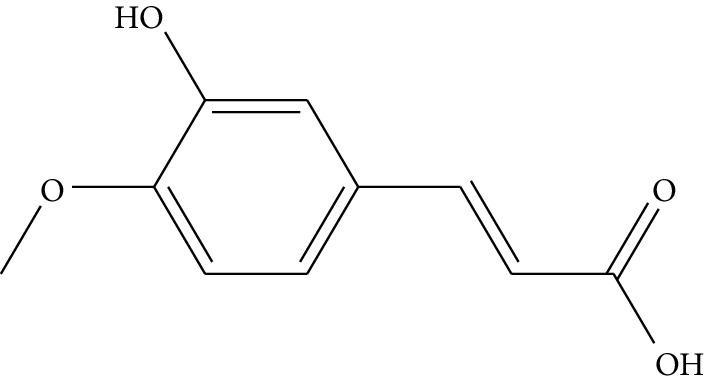	50.83	0.06	2.45	7	*Astragalus membranaceus* (Fisch) Bunge
mol36	Betulinic acid	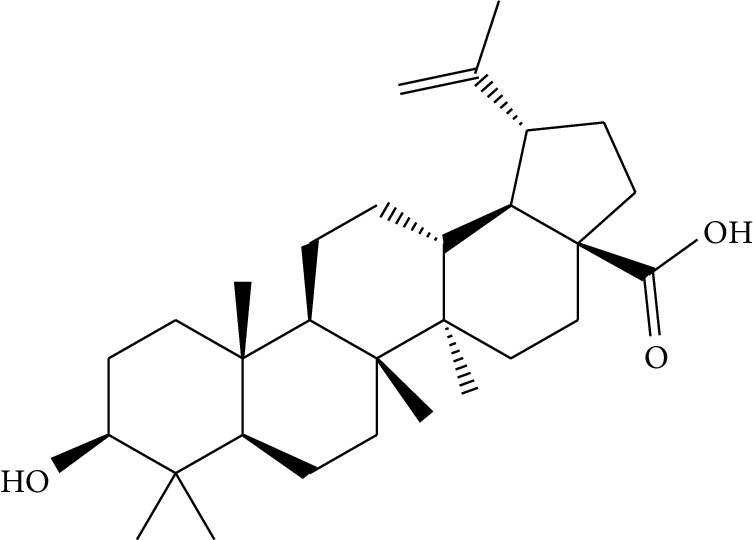	55.38	0.78	8.87	1	*Pulsatilla chinensis* (Bge.) Regel

mol37	Oleanolic acid	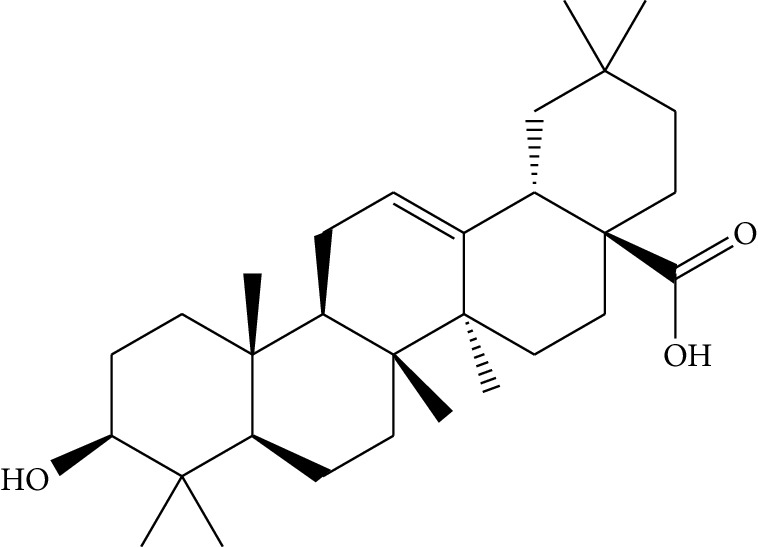	29.02	0.76	5.56	6	*Pulsatilla chinensis* (Bge.) Regel

mol38	Sitosteryl acetate	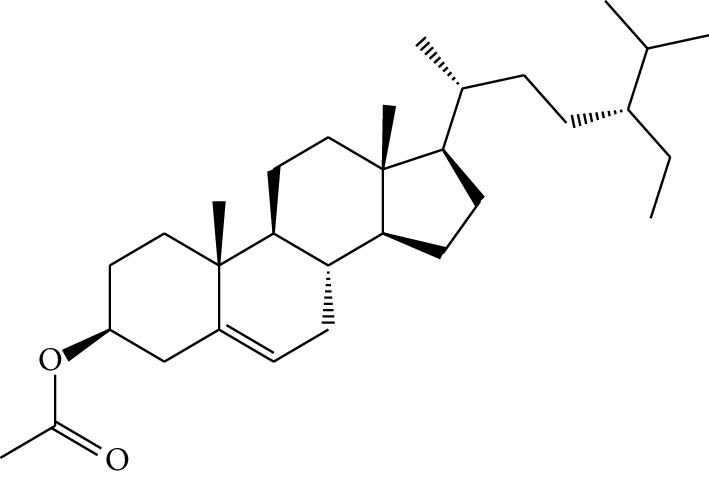	40.39	0.85	6.34	1	*Pulsatilla chinensis* (Bge.) Regel

mol39	Lanosterol	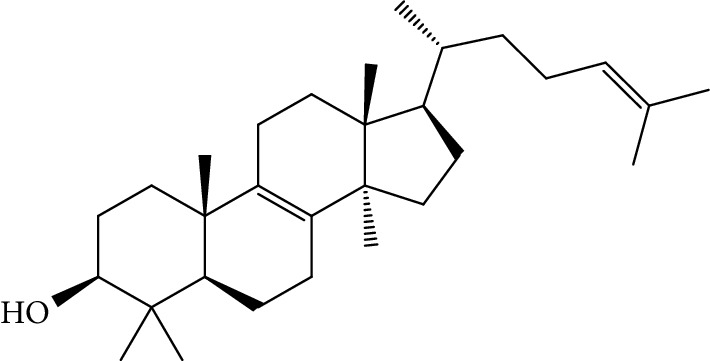	42.12	0.75	5.84	1	*Pulsatilla chinensis* (Bge.) Regel
mol40	3-beta,23-Dihydroxy-lup-20(29)-ene-28-O-alpha-L-rhamnopyranosyl-(1-4)-beta-D-glucopyranosyl(1-6)-beta-D-glucopyranoside_qt	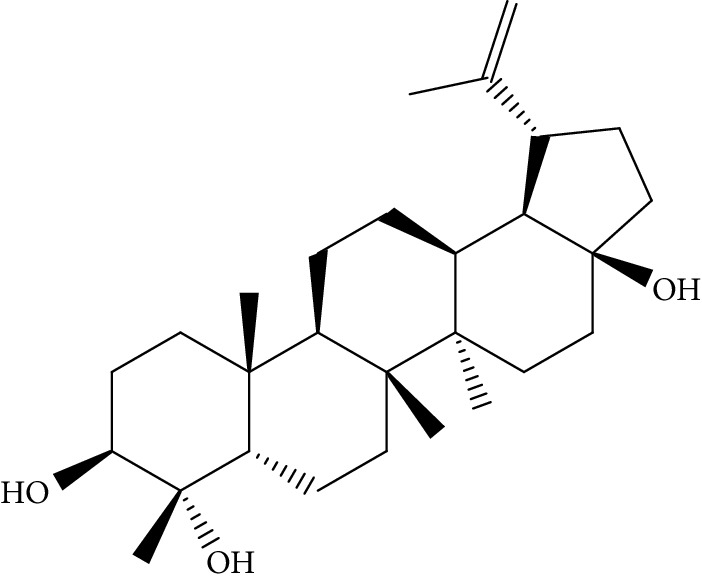	37.59	0.79	6.70	1	*Pulsatilla chinensis* (Bge.) Regel

mol41	Ursolic acid	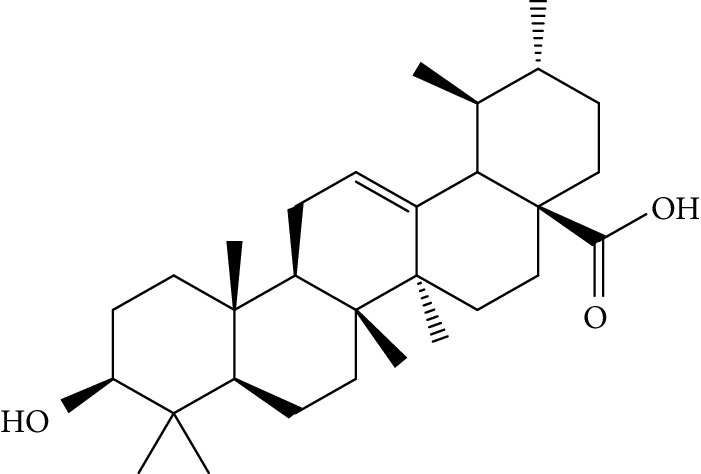	16.77	0.75	5.28	35	*Pulsatilla chinensis* (Bge.) Regel

**Table 2:** Details of 94 UC-related targets of herbs via UniProt.

ID	UniProt	Protein names	Gene names	Degree	Organism
1	P35228	Nitric oxide synthase, inducible	NOS2	25	Homosapiens
2	P23219	Prostaglandin G/H synthase 1	PTGS1	23	Homosapiens
3	P03372	Estrogen receptor	ESR1	34	Homosapiens
4	P37231	Peroxisome proliferator-activated receptor gamma	PPARG	21	Homosapiens
5	P35354	Prostaglandin G/H synthase 2	PTGS2	27	Homosapiens
6	Q92731	Estrogen receptor beta	ESR2	17	Homosapiens
7	P11388	DNA topoisomerase 2-alpha	TOP2A	5	Homosapiens
8	P16389	Potassium voltage-gated channel subfamily H member 2	KCNH2	13	Homosapiens
9	P08709	Coagulation factor VII	F7	6	Homosapiens
10	P29474	Nitric-oxide synthase, endothelial	NOS3	21	Homosapiens
11	P27338	Amine oxidase [flavin-containing] B	MAOB	5	Homosapiens
12	Q04206	Transcription factor p65	RELA	6	Homosapiens
13	P00533	Epidermal growth factor receptor	EGFR	1	Homosapiens
14	P31749	RAC-alpha serine/threonine-protein kinase	AKT1	2	Homosapiens
15	P15692	Vascular endothelial growth factor A	VEGFA	2	Homosapiens
16	P24385	G1/S-specific cyclin-D1	CCND1	3	Homosapiens
17	P10415	Apoptosis regulator Bcl-2	BCL2	5	Homosapiens
18	P01100	Proto-oncogene c-Fos	FOS	3	Homosapiens
19	P38936	Cyclin-dependent kinase inhibitor 1	CDKN1A	4	Homosapiens
20	P55211	Caspase-9	CASP9	4	Homosapiens
21	P00749	Urokinase-type plasminogen activator	PLAU	4	Homosapiens
22	P08253	72 kDa type IV collagenase	MMP2	2	Homosapiens
23	P14780	Matrix metalloproteinase-9	MMP9	2	Homosapiens
24	P22301	Interleukin-10	IL10	1	Homosapiens
25	P01133	Proepidermal growth factor	EGF	1	Homosapiens
26	P06400	Retinoblastoma-associated protein	RB1	2	Homosapiens
27	P01375	Tumor necrosis factor	TNF	6	Homosapiens
28	P05412	Transcription factor AP-1	JUN	4	Homosapiens
29	P05231	Interleukin-6	IL-6	3	Homosapiens
30	P42574	Caspase-3	CASP3	7	Homosapiens
31	P04637	Cellular tumor antigen p53	TP53	4	Homosapiens
32	P11926	Ornithine decarboxylase	ODC1	1	Homosapiens
33	Q14790	Caspase-8	CASP8	3	Homosapiens
34	P00441	Superoxide dismutase [Cu-Zn]	SOD1	2	Homosapiens
35	P17252	Protein kinase C alpha type	PRKCA	2	Homosapiens
36	P03956	Interstitial collagenase	MMP1	3	Homosapiens
37	P42224	Signal transducer and activator of transcription 1-alpha/beta	STAT1	2	Homosapiens
38	P04626	Receptor tyrosine-protein kinase erbB-2	ERBB2	1	Homosapiens
39	P09601	Heme oxygenase 1	HMOX1	3	Homosapiens
40	P05177	Cytochrome P450 1A2	CYP1A2	2	Homosapiens
41	P01106	Myc proto-oncogene protein	MYC	1	Homosapiens
42	P05362	Intercellular adhesion molecule 1	ICAM1	4	Homosapiens
43	P01584	Interleukin-1 beta	IL1B	5	Homosapiens
44	P13500	C-C motif chemokine 2	CCL2	1	Homosapiens
45	P19320	Vascular cell adhesion protein 1	VCAM1	2	Homosapiens
46	P10145	Interleukin-8	CXCL8	2	Homosapiens
47	P05771	Protein kinase C beta type	PRKCB	2	Homosapiens
48	O15392	Baculoviral IAP repeat-containing protein 5	BIRC5	2	Homosapiens
49	P04792	Heat shock protein beta-1	HSPB1	1	Homosapiens
50	P01137	Transforming growth factor beta-1	TGFB1	3	Homosapiens
51	P60568	Interleukin-2	IL2	2	Homosapiens
52	Q16678	Cytochrome P450 1B1	CYP1B1	2	Homosapiens
53	P00750	Tissue-type plasminogen activator	PLAT	1	Homosapiens
54	P01579	Interferon gamma	IFNG	4	Homosapiens
55	P09917	Arachidonate 5-lipoxygenase	ALOX5	3	Homosapiens
56	P60484	Phosphatidylinositol-3,4,5-trisphosphate 3-phosphatase and dual-specificity protein phosphatase PTEN	PTEN	1	Homosapiens
57	P05164	Myeloperoxidase	MPO	1	Homosapiens
58	Q9UNQ0	ATP-binding cassette subfamily G member 2	ABCG2	1	Homosapiens
59	P09211	Glutathione S-transferase P	GSTP1	3	Homosapiens
60	Q16236	Nuclear factor erythroid 2-related factor 2	NFE2L2	1	Homosapiens
61	P15559	NAD(P)H dehydrogenase [quinone] 1	NQO1	2	Homosapiens
62	P09874	Poly [ADP-ribose] polymerase 1	PARP1	1	Homosapiens
63	P35869	Aryl hydrocarbon receptor	AHR	2	Homosapiens
64	P19875	C-X-C motif chemokine 2	CXCL2	1	Homosapiens
65	O96017	Serine/threonine-protein kinase Chk2	CHEK2	1	Homosapiens
66	Q07869	Peroxisome proliferator-activated receptor alpha	PPARA	1	Homosapiens
67	P02741	C-reactive protein	CRP	1	Homosapiens
68	P02778	C-X-C motif chemokine 10	CXCL10	1	Homosapiens
69	Q9NS23	Ras association domain-containing protein 1	RASSF1	1	Homosapiens
70	P17936	Insulin-like growth factor-binding protein 3	IGFBP3	1	Homosapiens
71	P01344	Insulin-like growth factor II	IGF2	1	Homosapiens
72	P21860	Receptor tyrosine-protein kinase erbB-3	ERBB3	1	Homosapiens
73	P09488	Glutathione S-transferase Mu 1	GSTM1	2	Homosapiens
74	P28223	5-Hydroxytryptamine 2A receptor	HTR2A	4	Homosapiens
75	P84022	Mothers against decapentaplegic homolog 3	SMAD3	1	Homosapiens
76	P08588	Beta-1 adrenergic receptor	ADRB1	3	Homosapiens
77	P29466	Caspase-1	CASP1	2	Homosapiens
78	P18509	Pituitary adenylate cyclase-activating polypeptide	ADCYAP1	1	Homosapiens
79	O95456	Proteasome assembly chaperone 1	PSMG1	1	Homosapiens
80	P05112	Interleukin-4	IL-4	1	Homosapiens
81	P00325	Alcohol dehydrogenase 1B	ADH1B	1	Homosapiens
82	P28702	Retinoic acid receptor RXR-beta	RXRB	1	Homosapiens
83	P04040	Catalase	CAT	1	Homosapiens
84	P01308	Insulin	INS	1	Homosapiens
85	P21731	Thromboxane A2 receptor	TBXA2R	1	Homosapiens
86	P07858	Cathepsin B	CTSB	1	Homosapiens
87	P40763	Signal transducer and activator of transcription 3	STAT3	1	Homosapiens
88	Q00534	Cell division protein kinase 6	CDK6	1	Homosapiens
89	P09038	Heparin-binding growth factor 2	FGF2	1	Homosapiens
90	P15336	Cyclic AMP-dependent transcription factor ATF-2	ATF2	1	Homosapiens
91	P04141	Granulocyte-macrophage colony-stimulating factor	CSF2	1	Homosapiens
92	P17677	Neuromodulin	GAP43	1	Homosapiens
93	P18031	Tyrosine-protein phosphatase nonreceptor type 1	PTPN1	1	Homosapiens
94	P30279	G1/S-specific cyclin-D2	CCND2	1	Homosapiens

## Data Availability

The datasets used and analyzed during the current study are available by sending email to the corresponding author.
